# Influence of pharmaceutical marketing mix strategies on physicians’ prescribing behaviors in public and private hospitals, Dessie, Ethiopia: a mixed study design

**DOI:** 10.1186/s12889-020-10063-2

**Published:** 2021-01-07

**Authors:** Abel Demerew Hailu, Birhanu Demeke Workneh, Mesfin Haile Kahissay

**Affiliations:** 1 Department of Pharmacy, Dessie Health Science College, Dessie, Ethiopia; 2 Department of Pharmacy, College of Medicine and Health Science, Wollo University, Dessie, Ethiopia

**Keywords:** Pharmaceutical, Marketing mix, Physician, Prescribing behavior

## Abstract

**Background:**

Prescription drugs constitute the primary source of revenue for the pharmaceutical industry. Most pharmaceutical companies commit a great deal of time and money to market in hopes of convincing physicians about their products. The objective of this study is to assess perceived influence of pharmaceutical marketing mix strategies on physicians’ prescribing behaviors in hospitals, Dessie, Ethiopia.

**Methods:**

Mixed methods sequential explanatory design was employed in two public and three private hospitals. A cross-sectional study design was employed by including (136) physicians working in public and private hospitals. Percentage, mean, standard deviation, and multiple linear regressions were computed using Statistical Package for Social Science. In the second phase, the phenomenological design was employed to fully explore in-depth information. Purposive sampling was used to select key informants and 14 in-depth interviews were conducted by the principal investigator. Content analysis was performed using Nvivo 11 plus and interpretation by narrative strategies.

**Results:**

The overall perceived influence of pharmaceutical marketing mix strategies in physicians’ prescribing behavior was 55.9%. The influence of promotion, product, place and price strategy perceived by physicians in their prescribing behavior was 83 (61%), 71(52.2%), 71 (52.2%), 80 (58.8%) respectively. There was a statistically significant difference among marketing mix strategies (β = 0.08, *p* = < 0.001). Determinants on the influence of physicians’ prescribing behavior were specialty (*p* = 0.01) and working areas (*p* = 0.04). The qualitative design also generates additional insights into the influence of pharmaceutical marketing mix strategies on physician prescribing behavior.

**Conclusions:**

More than half of physicians perceived that pharmaceutical marketing mix strategies influence their prescribing behavior. The qualitative design also revealed that pharmaceutical marketing mix strategies influenced physicians prescribing behavior. Strengthening the regulation and maintaining ethical practice would help to rationalize the physicians’ prescribing practice.

**Supplementary Information:**

The online version contains supplementary material available at 10.1186/s12889-020-10063-2.

## Background

According to Philip Kotler and Armstrong marketing is defined as “Satisfying needs and wants through an exchange process” [1]. The pharmaceutical marketing mix first introduced by Borden in 1964 with the basic elements are the product, price, place, and promotion (collectively coined the 4Ps of marketing) [2]. 4Ps are linked to each other to generate a prescription order by physicians and makes the product reaches to the consumers [3] this creates for a firm to get the desired level of sales in the target market [4].

Marketing prescription medicines constitute the primary source of revenue/profits for the pharmaceutical company [5]. Pharmaceutical companies commonly employ a wide range of marketing strategies to increase their drug sales [6, 7]. Eighty four percent of pharmaceutical marketing efforts are directed toward physicians because from the manufacturer’s point of view, physicians are the gatekeepers or decision-makers to drug sales [8]. Most pharmaceutical manufacturer and distributor companies commit a great deal of time and money to market in hopes of convincing physicians about their products [9].

Regardless of codes of pharmaceutical medical practice [10] and World Health Organization code of ethics regulating the marketing of prescription drugs, there are still unethical commercial practices which influence prescribers’ decisions [11] by supporting with biased information and more engaged in creating higher profits to the company [12].

Marketing the pharmaceutical industry is a large and high-value industry in the globe, where its practices have a direct influence on the welfare of patients at the individual level and society in general [13]. The different company utilizes many ways in marketing their product such as giving away gifts, free lunches, incentives, sponsoring education and holidays as inducements which compel a doctor to prescribe without a scientific basis [14].

Ethiopia is one of the most populous countries in Africa and the demand for pharmaceutical products in the country is high [15]. The manufacturing of pharmaceutical is quite small and covers between 10 and 20% of the domestic market and the rest of the market are satisfied through imports [16]. In 2015, the annual pharmaceutical market in Ethiopia was estimated at United States of America Dollar (US$) 400 to 500 Million and expected to reach around US$ 1 billion by 2018 [17]. According to Ethiopian food and drug administration (EFDA), regulation directive marketing of pharmaceutical products is restricted and a retailer or wholesaler cannot market prescription products or services directly to the end consumer [18].

District hospital, health centers, and health post facilities found in adjacent zones areas of Dessie city rather than referral and general hospitals. This infrastructure and national treatment guideline limit the health officers to diagnosed and treat patients had different disease. Due to that, a significant number of patients have been visiting Dessie city for seeking health services. Thus some public and private health institutions are concentrated. Taking this advantage the number of pharmaceutical distributors/wholesalers in Dessie town has been increasing steadily over the years. In some streets of the town, a very high concentration of pharmaceutical service providers is found to the extent that five licensed premises within the same building or a walking distance of each other. This scenario necessarily elicits a high degree of market competition among various companies. In the absence of such direct marketing, the players have to find innovative ways to stand out of the crowd and beat the competition because of the existence of various brands of generic medications [19]. The competitive nature of the market triggers a pharmaceutical company to develop pharmaceutical marketing strategies to convince physicians to beat the competitor and to get reasonable profits.

As pharmaceutical spending continues to escalate and drug safety issues have become more common, such physician-directed outreach efforts have come under mounting public scrutiny [20]. Pharmaceutical firms, therefore, need to design their marketing mixes strategies without affecting the ethical code of practice. They need to understand how their marketing mixes influence the doctors’ choice of prescription drugs. So far limited research had been carried out about the influence of pharmaceutical marketing mix strategies on physicians prescribing behaviors [21, 22]. Therefore, the objective of this study was to assess perceived influence of pharmaceutical marketing mix strategies on physicians’ prescribing behaviors in hospitals, Dessie, Ethiopia.

## Methods

### Study area and period

A study was conducted from September 1 – October 1, 2019 at private and public hospitals, Dessie, Ethiopia. Dessie is located in South Wollo zone of Amhara Regional State, 401 km away from Addis Ababa, the capital city of Ethiopia.

### Study design

Mixed methods sequential explanatory design was employed. A first phase is begun by a cross-sectional study design to assess perceived influence of pharmaceutical marketing mix strategies on physicians’ prescribing behavior. This was followed by a phenomenological design used to fully explore in-depth information about underlining reasons, opinions and motivational behaviors of physicians.

### Source and study population

All physicians working in public and private hospitals were found in Dessie city considered as a source population. Physicians who were available and volunteer to participate during the study period were included in the study.

### Sample size determination and sampling procedures

For quantitative part of the study, all physicians (140) working in public and private hospitals of Dessie town were included, by consulting human resource department of Dessie city health office, so the issue of representativeness of the population was guaranteed. Key informants were selected by the principal investigator by screening the doctors that medical representatives (MRs) often target; i.e. using MRs, as gatekeeper, who work in Dessie city pointed out highly experienced and targeted physicians, and by their patient load (from the patient data clerk registration form of each hospital). Purposive sampling was used to select physicians working in public and private hospitals as key informants for qualitative part of the study since they are supposed to be experienced and rich in information related to pharmaceutical marketing activities performed by pharmaceutical companies. The number of the key informants was 14 due to the saturation of information concerning emerging themes.

### Data collection instruments and procedures

A structured questionnaire (see Additional file 1) was developed from literatures [21, 23–25] to measure the influence of pharmaceutical marketing mix strategies, such as pricing, promotional, places, product strategies, and socio-demographic characteristics of physicians on physicians’ prescribing behavior. The questionnaires, which were delivered to the participants in person, included demographic questions and five width Likert-style questions (44 questions). The Likert-question answers ranged from “strongly disagree” to “strongly agree”. The quantitative data was collected by 5 nurses using data collection self-administered questionnaires after recruiting and half-day training. The principal investigator coordinated data collection.

An interview guide (see Additional file 2) was utilized for the qualitative part to assess in depth-related information on the behavior of physicians towards how pharmaceutical marketing mix elements such as promotion, products, places, and price strategy affects their prescribing behavior. This was conducted through face to face interviews with physicians, by the principal investigator (ADH) using open-ended questions, on hospital compound and public places, such as cafés and hotels. The interview guide was organized and developed from reputable literatures [21, 23–25]. The principal investigator conducted an in-depth interview which lasted an average of 38 min (25 to 59 min) and until no new theme emerged. The interview was conducted in Amharic, official language of Ethiopia, with the aid of the audio recorder. All physicians, natives to Ethiopia, and fluently speaks the Amharic language to explain their experience. The principal investigator took notes during the interview. In cases of ambiguity, the principal investigator clarifies the issues raised instantaneously. All interviews recorded were transcribed to a verbatim.

### Reflexivity: the principal investigator status as an insider

The principal investigator’s status as a “professional” and “native” offers certain strengths and insights into the professional issues he was exploring. The principal investigator was non-judgmental while in the in-depth interview and maintained professional relativity. He considered insider bias in his work and justified how other key informants respond to him. He was also faced with the challenge based on his position as a member of the elite and senior pharmacy professional.

All of these issues concerning competing roles and perceptions related to the concept of insider bias, which has both advantages and disadvantages when conducting such a study. In his case, the advantages included being able to use existing networks and contacts within the physician, than might otherwise have been available to him. On the other hand, the disadvantages related to his position include the way he was perceived by the participants in this study; it is impossible to know the extent to which his participants were truthful in the perceptions and opinions they share with him or whether they were telling him the things they think he wants to hear. The use of open-ended questions, as well as efforts made to engage informants in informal conversations on other topics, they themselves raised, were among some of the measures taken to mitigate these limitations.

### Data quality management

For the quantitative survey self-administered questionnaire was prepared in English. Interview guide was prepared in English, translated into Amharic and finally back-translated into English to maintain consistency and standardization of the instruments. To assure the quality of the data, the self-administered questionnaire tool was properly designed and its reliability was checked by the Cronbachs Alpha test (0.94). Validity of questionnaire was assured by using standardized questionnaire adapted from literatures [21, 23, 25]. Also, the principal investigator and supervisors closely monitor the data collection process. The assumption of linear regression (independent observation, normal distribution and homogeneity of variance) test was fulfilled. Any unfilled data in the questionnaire was first checked and all collected data were critically examined for completeness and consistency during data collection, analysis and, interpretation. The pre-test was carried out to test study instruments with thirty-five physicians in a health facility that was not part of the study area. Sensitive issues to physicians like ethnicity and salary questions were removed from the questionnaire after conducting the pre-test. Also, different strategies were used to assure the quality of the data: theoretical, using conceptual frameworks to guide the study, a mixed approach (qualitative and quantitative), and more than one investigator involved in this study. Also, the qualitative findings were shared with key informants to confirm the presentations accurately reflected their perceptions and experiences. The content of the interview guide was checked by an expert, from a social and administrative pharmacy department group.

### Data analysis and presentation

EpiData software (version 4.6.0) was utilized for coding and data entry processes after the data edited. The quantitative data were analyzed using the Statistical Package for Social Sciences (SPSS) version 20. Simple descriptive analysis such as percentage, mean and standard deviation (SD) was computed to meet the stated objective. Microsoft excel program was used to present summary results in terms of figures and tables. Also, inferential statistics were computed using multiple linear regressions to measure the association between independent and dependent variables with a 95% confidence interval and variable with *p*-value < 0.05 taken as statistically significant.

Early coding concurrently with data collection was conducted on audio-recorded and transcribed. Data were analyzed using the principles of inductive content analysis. Texts were read independently by the principal investigator (ADH) and another professional who speaks the local language (MHK) and codes were developed in reference to the research questions. Each of the codes were organized into higher-order conceptual themes. These individual codes and themes were discussed at group meetings until consensus was reached on basic themes and subthemes across interviews. Finally, the themes were incorporated into a conceptual model of the participants and the influence of pharmaceutical marketing mix strategies on prescribing behaviors of physicians. Sections of original transcripts and key quotes considered to be illustrative of the emerging themes were translated into English to facilitate discussion with the full research team. Data analysis was supported by the use of NVivo 11 plus computer software Narrative strategies was employed for interpretation and the identifier codes for the presentation of quotations in the qualitative findings were: specialty, working areas, and age of key informants.

### Operational definitions

- **Influenced**: Physicians scored greater or equal to mean score for pharmaceutical marketing mix strategy was influenced.
- **Not-influenced**: Physicians scored below mean score for pharmaceutical marketing mix strategy was not influenced.

## Results

### Socio-demographic characteristics of respondents

A total of 136 questioners were collected during data collection period with the response rate 97.14%. As shown in Table 1, 120 (88.2%) of the respondents in the study were male and 16 (11.8%) were female. The majority of respondents 49 (36%) were between the age of 30–44 years. Looking at the specialty of participants, 20(26.3%) were residents, 16(21.1%) were internists and 12(15.8%) were surgeons.

**Table 1:** Socio-demographic characteristics of respondents in hospitals of Dessie, Ethiopia (*n* = 136)

No.	Variables	Categories	Frequency	Percent
1	Sex	Male	120	88.2
		Female	16	11.8
2	Age	25–29	35	25.7
		30–34	49	36
		35–39	27	19.9
		> = 40	25	18.4
3	Education	General practitioners	60	44.1
		Specialized	76	55.9
4	Specialty	Internist	16	21.1
		Surgeon	12	15.8
		Gynecologist	10	13.2
		Pediatrician	6	7.9
		Dermatologist	3	3.9
		Orthopedics	3	3.9
		Resident	20	26.3
		ENTs	6	7.9
5	Country of specialization	Ethiopia	74	97.3
		Outside Ethiopia	2	2.7
6	Working experience	< 5 years	84	61.8
		5–10 years	26	19.1
		> 10 years	26	19.1
7	Working areas	Public	84	61.8
		Private	18	13.2
		Both	34	25

### Participants for qualitative section

A total of 14 professionals participated in in-depth interviews. All key informants were male and within the age of 32–45 with mean and SD 39.86 ± 3.86. Regarding the place of work, 5 interviewees were from public health facilities and 9 were from private health facilities. Key informants included were 3 internists, 3 surgeons, 2 pediatrician, 2 gynecologists, 2 orthopedician, and 2 GPs. The work experience of key informants ranges from 4 to 15 years with mean and SD 10.07 ± 2.94.

### Influence of promotion strategy on physicians prescribing behavior

Physicians perceived that information from MRs (3.85 ± 1.11), participating in company-sponsored CME (3.61 ± 1.2), participating product launch meeting (3.5 ± 1.01), frequent visits of MRs (3.64 ± 1.08), information from promotional drug brochures (3.47 ± 1.11), and invitation to visit a pharmaceutical manufacturing plant (3.52 ± 1.22) were influenced their prescribing behavior. However, promotional strategy tools like receiving different gifts from pharmaceutical company (3.09 ± 1.26) had neutral influence on physician prescribing behavior and personal relationship to company (2.74 ± 1.11) did not influence their prescribing behavior (Table 2).

**Table 2:** Perceived influence of promotion strategy on physicians’ prescribing behavior in hospitals of Dessie, Ethiopia (*n* = 136)

No.	Description	SD^a^ N (%)	D^a^ N (%)	N^a^ N (%)	A^a^ N (%)	SA^a^ N (%)	Mean ± SD
1	Participating in company-sponsored continual medical education	13 (9.6)	13(9.6)	18 (13.1)	62 (45.6)	30 (22.1)	3.61 ± 1.20
2	Information from medical representative	7(5.1)	12 (8.8)	17 (12.6)	58 (42.6)	42 (30.9)	3.85 ± 1.11
3	Frequent visits of medical representative	5 (3.7)	19 (14)	25 (18.4)	58 (42.6)	29 (21.3)	3.64 ± 1.08
4	Sales calls made by pharmaceutical companies	7 (5.1)	33 (24.3)	45 (33.1)	40 (29.4)	11 (8.1)	3.11 ± 1.03
5	Free drug samples given by pharmaceutical company	15 (11)	16 (11.8)	24 (17.7)	63 (46.3)	18 (13.2)	3.39 ± 1.18
6	Information from promotional drug brochures	8 (5.9)	20 (14.7)	31 (22.8)	54 (39.7)	23 (16.9)	3.47 ± 1.11
7	Different gifts from pharmaceutical company	22 (16.2)	22 (16.2)	28 (20.5)	50 (36.8)	14 (10.3)	3.09 ± 1.26
8	Participating pharmaceutical company-sponsored entertainments/recreational event	16 (11.8)	23 (16.9)	23 (16.9)	53 (39)	21 (15.4)	3.29 ± 1.25
9	Sponsorship for travel in conference	11 (8.1)	21(15.4)	32 (23.5)	44 (32.4)	28 (20.6)	3.42 ± 1.20
10	Subscription of journals with direct mail	11 (8.1)	21(15.4)	22 (16.2)	56 (41.2)	26 (19.1)	3.48 ± 1.19
11	Invitation to visit a pharmaceutical manufacturing plant	11 (8.1)	20 (14.7)	23 (16.9)	51 (37.5)	31 (22.8)	3.52 ± 1.22
12	Personal relationship to company	21 (15.4)	35 (25.7)	46 (33.9)	26 (19.1)	8 (5.9)	2.74 ± 1.11
13	Participating to product launch meeting	5 (3.7)	20 (14.7)	31 (22.8)	62 (45.6)	18 (13.2)	3.50 ± 1.01
14	Public relation of pharmaceutical company	9(6.6)	23 (16.9)	36 (26.5)	42 (30.9)	26 (19.1)	3.39 ± 1.16

^a^Responses ranged from strongly disagree (1) to strongly agree (5)

The qualitative part of study found that pharmaceutical companies follow a variety of strategies as they seek to further increase their market share in Dessie town. They are trying to find doctors by adopting a pharmaceutical marketing mix strategy. One of those used a strategies was promotion. Pharmaceutical companies will hire MRs in the town to get the physicians closer to promote their products and to explain the company’s image. Also, those who do not have a MRs here will send their MRs from the capital city for some time and maintain close ties with the physicians. The majority of participants (*n* = 10) in the qualitative study reported that receiving medication information through MRs and seeing the manufacturing site had a positive influence on their prescriptions pattern. One of an informant described this scenario:

“If you take MRs with me now … … if you find them somewhere else, what is the whole thing? That product will bring to your mind the product that promoter will introduce to you and have influence here and these things will affect my prescription order (Orthopedician, Public, 40).”

In addition, this was emphasized by another informant:

“… ..seeing where the pharmaceutical products are manufactured is also very interesting to me, I have personally gone twice to saw pharmaceutical manufacturing site, has led to me an increase in the prescribing rate of drugs.....(Internist, Private, 41).”

### Perceived influence of place strategy on physicians prescribing behavior

Respondents perceived that pharmaceutical product availability (4.26 ± 0.77), the inclusion of the medicine in the hospital medicine list (3.79 ± 0.98), fast deliveries with special storage and distribution of medicines (3.66 ± 1.08), availability of real-time product information from distribution intermediaries (3.61 ± 1.11) and availability of local agents (importer/distributor) representing the principal company (3.4 ± 1.14) were influenced physicians when prescribing medication to the patients (Table 3).

**Table 3:** Perceived influence of place strategy on physicians’ prescribing behavior in hospitals of Dessie, Ethiopia (*n* = 136)

No.	Description	SD^a^ N (%)	D^a^ N (%)	N^a^ N (%)	A^a^ N (%)	SA^a^ N (%)	Mean ± SD
1	Pharmaceutical product availability	0 (0)	6 (4.4)	10 (7.4)	63(46.3)	57 (41.9)	4.26 ± 0.77
2	Inclusion of medicine in the hospital medicine list	1 (0.7)	14 (10.3)	34 (25)	50(36.8)	37 (27.2)	3.79 ± 0.98
3	Availability of local agent (importer/distributor) representing the principal company	7 (5.1)	25 (18.4)	36 (26.5)	42 (30.9)	26 (19.1)	3.40 ± 1.14
4	Availability of real-time product information from distribution intermediaries	4 (2.9)	24 (17.6)	25 (18.5)	51 (37.5)	32 (23.5)	3.61 ± 1.11
5	Presence of sole supplier	6 (4.4)	31 (22.8)	39 (28.6)	50 (36.8)	10 (7.4)	3.20 ± 1.01
6	Fast deliveries with special storage and distribution of medicines	3(2.2)	22 (16.2)	26 (19.1)	52 (38.2)	33 (24.3)	3.66 ± 1.08
7	Reverse pharmaceutical (product recall)	8 (5.9)	24 (17.6)	47 (34.6)	37 (27.2)	20 (14.7)	3.27 ± 1.09

^a^Responses ranged from strongly disagree (1) to strongly agree (5)

A pharmaceutical company use place strategy to expand its market share by persuading physicians to prescribe their products. Most (*n* = 10) of those who participated in the qualitative study emphasized that the companies are focused on improving the supply chain performance of pharmaceuticals has made their medical work easier. This was further substantiated by one of an informant:

“If you are prescribing some drugs, they will bring that product and the primary benefit for the patient. A patient does not send the prescription to Addis Ababa they easily get here within a variety of pharmacies in the city is such a great job for us (Pediatrician, Private, 44).”

One of the place strategies was having an agent in the city of Dessie made it easy for them to prescribe medications for patients. This was supported by one informant from the public sector revealed that:

“… .to be honest, I don’t want to roam a patient in the city. If we have these medications, we can prescribe medications at the pharmacy and I don’t prescribe the medicine if it’s not available outside the pharmacy. There is no need to wander the patient (Gynecologist, Public, 41).”

### Perceived influence of price strategy on physicians prescribing behavior

Regarding price strategy dimensions, respondents perceived that the price of the drug and effectiveness of therapy (4.13 ± 0.89), disclosure of actual price of the product (3.87 ± 0.97), price discounts technique for the product (3.71 ± 1) and price of medication to quality (3.72 ± 1.07) were influence on physicians’ prescribing behavior (Table 4).

**Table 4:** Perceived influence of price strategy on physicians’ prescribing behavior in hospitals of Dessie, Ethiopia (*n* = 136)

No	Description	SD^a^ N (%)	D^a^ N (%)	N^a^ N (%)	A^a^ N (%)	SA^a^ N (%)	Mean ± SD
1	Disclosure of actual price of the product	3 (2.2)	8 (5.9)	32 (23.5)	54 (39.7)	39 (28.7)	3.87 ± 0.97
2	Price discounts technique for the product	1 (0.7)	19 (14)	31 (22.8)	53 (39)	32 (23.5)	3.71 ± 1.00
3	Price of the drug and effectiveness of therapy	1 (0.7)	9 (6.6)	14 (10.3)	60 (44.1)	52 (38.2)	4.13 ± 0.89
4	Price of medication in relation to quality	2 (1.5)	20 (14.7)	30 (22.2)	46 (33.8)	38 (27.9)	3.72 ± 1.07
5	Price competition among pharmaceutical company	6 (4.4)	24 (17.6)	46 (33.9)	38 (27.9)	22 (16.2)	3.34 ± 1.08
6	Price for full course therapy	10 (7.4)	14 (10.3)	22 (16.1)	61 (44.9)	29 (21.3)	3.63 ± 1.14

^a^Responses ranged from strongly disagree (1) to strongly agree (5)

Pharmaceutical companies utilize strategy to attract the attention of physicians to maintain their market status and increase their revenues. Most of key informants (*n* = 8) recognizing the cost of the drug had a benefit and a positive influence on their work when prescribing medication. This was supported by one of an informant that:

“....knowing the product price makes us comfortable with prescribing a product in the first place. This is important to identify individuals who have potential or users can use a product (GPs, Public, 34).”

### Perceived influence of product strategy on physicians prescribing behavior

As shown in Table 5, physicians perceived that supportive evidence of the efficacy of the medicine given by the pharmaceutical company (4.01 ± 0.86), the release of new innovations or combinations of drugs (4.07 ± 0.97), quality of the medicine (4.12 ± 0.98), form of delivery of the medicine (3.88 ± 0.95), introducing the country of manufacturer of pharmaceutical products (3.6 ± 1.06), and the image of a pharmaceutical company (3.51 ± 1.08) were influenced physicians’ prescribing behavior.

**Table 5:** Perceived influence of product strategy on physicians’ prescribing behavior in hospitals of Dessie, Ethiopia (*n* = 136)

No.	Description	SD^a^ N (%)	D^a^ N (%)	N^a^ N (%)	A^a^ N (%)	SA^a^ N (%)	Mean ± SD
1	Country of pharmaceutical product manufacturer	6 (4.4)	14 (10.3)	36 (26.5)	52 (38.2)	28 (20.6)	3.60 ± 1.06
2	Image of pharmaceutical company	4 (2.9)	24 (17.6)	33 (24.4)	49 (36)	26 (19.1)	3.51 ± 1.08
3	Supportive evidence of the efficacy of the medicine given by pharmaceutical company	0 (0)	10 (7.4)	20 (14.6)	64 (47.1)	42 (30.9)	4.01 ± 0.86
4	Release of new innovations or combinations of drugs	1 (0.7)	12 (8.8)	17 (12.6)	52 (38.2)	54 (39.7)	4.07 ± 0.97
5	Form of delivery of the medicine	1 (0.7)	15 (11)	20 (14.7)	64 (47.1)	36 (26.5)	3.88 ± 0.95
6	Easy to remember brand names	9 (6.6)	22 (16.2)	31 (22.8)	55 (40.4)	19 (14)	3.39 ± 1.11
7	Reputation of the source of medicine	9 (6.6)	16 (11.8)	42 (30.8)	50 (36.8)	19 (14)	3.40 ± 1.07
8	Quality of medicine	2 (1.5)	11 (8.1)	13 (9.5)	53 (39)	57 (41.9)	4.12 ± 0.98
9	Fixed-dose packaging of the product	5 (3.7)	18 (13.2)	21 (15.5)	52 (38.2)	40 (29.4)	3.76 ± 1.12
10	Full therapy packaging	8 (5.9)	24 (17.6)	26 (19.1)	51 (37.5)	27 (19.9)	3.48 ± 1.16

^a^Responses ranged from strongly disagree (1) to strongly agree (5)

A pharmaceutical manufacturer or importer companies try to attract doctors’ attention to increase their market share in the Dessie town. Most (*n* = 9) of the participants in the qualitative study agreed that the change in the preparation of the drug formulations has a positive effect on when they prescribe medicines to their patients. Regarding this an informant had to say:

“… … a combination that comes with a new look. For example, if we take Aferin now, this combination of three drugs is very good for me, to prescribe for the patient. You can make it easier to deliver, especially at pediatrics age and you will reduce overload (Pediatrician, Private, 38).”

The study also found that the country of origin had influence on their prescribing behavior. In this regard majority (*n* = 9) of informants think that the drug has a good quality and they said it has a positive effect on their work when prescribing medicine to patients. To this, one of the key informants stated that:

“… ..knowing about the companies is a good idea. It has influence sometimes; I think it is better to know what country the products are from if you know the country you should decide what to do (Gynecologist, Public, 41).”

### Perceived influence of marketing strategies

**Fig. 1:**
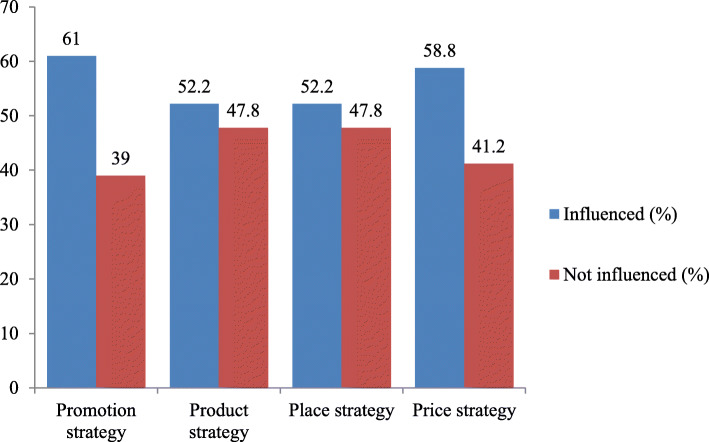
Marketing mix perceived influence on prescribing behavior in hospitals of Dessie, Ethiopia

The data fulfilled the assumption of normal distribution, the mean score was appropriate to classify the influence of pharmaceutical marketing mix strategies on physicians’ prescribing behavior. Accordingly, promotional strategies, product strategies, place strategies, and price strategies were reported with the overall mean score of 3.39, 3.72, 3.59 and 3.73 respectively. Respondents’ scores above this mean score were influenced. As shown in Fig. 1, regarding marketing mix elements; promotion strategy, product strategy, place strategy and price strategy 83(61%), 71(52.2%), 71(52.2%), 80(58.8%) of physicians perceived that influenced their prescribing behavior respectively.

Seventy-six (55.9%) of respondents scored above the overall mean score and perceived that their prescribing behavior was influenced. Whereas, 60 (44.1%) of participants scored below the overall mean perceived that not influenced their prescribing behavior by pharmaceutical marketing mix strategies.

In addition, in the qualitative part of the study majority of participants (*n* = 10) reported that promoting of drug products by multinational companies had a positive influence on their prescriptions pattern. This was emphasized by one of an informant:

“Promotion of dugs I think it has to use update our knowledge, it has great importance for getting new things done (Internist, Private, 39).”

The Post Hoc test revealed that promotional strategies have a statistically significant difference with product (*p* = 0.001), place (*p* = 0.0028) and price (*p* = 0.00). There was a statistically significant difference among marketing mix strategies employed by pharmaceutical companies to influence physician prescribing behavior (β = 0.08, *p* = < 0.001) (Table 6).

**Table 6:** Linear regression among marketing strategies in hospitals of Dessie, Ethiopia

No.	Marketing mix strategies	Influenced N (%)	Not influenced N (%)	*P*-value	β (95% CI)
1	Product strategy	71(52.2)	65(47.8)	0.00	0.08 (0.03–0.14)
2	Place strategy	71(52.2)	65(47.8)		
3	Price strategy	80(58.8)	56(41.2)		
4	Promotion strategy	83(61)	53(39)		

The current study also identified, specialty (β = − 0.06, *p* = 0.01) and working areas (β = 0.15, *p* = 0.04) had a statically significant influence on physicians’ prescribing behavior but sex, age, education, country of first-degree education, country of specialty, and working experience were not found statistically significant (*p* > 0.05) (Table 7).

**Table 7:** Multiple linear regression of socio-demographic characteristics on physicians’ prescribing behavior in hospitals of Dessie, Ethiopia

No.	Variables	Categories	N (%)	*P*-value	β (95% CI)
1	Sex	Male	120 (88.2)	0.42	−0.17 (−0.61–0.25)
		Female	16 (11.8)		
2	Age	25–29	35 (25.7)	0.20	−0.11 (−0.29–0.06)
		30–34	49 (36)		
		35–39	27 (19.9)		
		> = 40	25 (18.4)		
3	Education	GPs	60 (44.1)	0.48	− 0.34 (−1.3–0.62)
		Specialized	76 (55.9)		
4	Specialty	Internist	16 (21.1)	0.01	−0.06 (−0.11- (− 0.01))
		Surgeon	12 (15.8)		
		Gynecologist	10 (13.2)		
		Pediatrician	6 (7.9)		
		Dermatologist	3 (3.9)		
		Orthopedics	3 (3.9)		
		Resident	20 (26.3)		
		ENTs	6 (7.9)		
5	Country of specialization	Ethiopia	74 (97.3)	0.82	0.07 (−0.61–0.77)
		Outside Ethiopia	2 (2.7)		
6	Working experience	< 5 years	84 (61.8)	0.38	− 0.1 (− 0.33–0.12)
		5–10 years	26 (19.1)		
		> 10 years	26 (19.1)		
7	Working areas	Public	84 (61.8)	0.04	0.15 (0.00–0.31)
		Private	18 (13.2)		
		Both	34 (25)		

## Discussion

Medicines are important components of the health care system and play a crucial role in saving a life. When used rationally, they produce the desired effect of improving patients’ ailments [26]. The main aim of physicians is to render service to patients in a rational manner [27]. The interaction between physician and pharmaceutical companies must be ethical if a proper medication prescribing practice is required.

In this study, the perceived influence of pharmaceutical marketing mix strategies on physicians’ prescribing behavior in Dessie town was 55.9%. This might be attributed to pharmaceutical sectors are more likely densely accumulated to take advantage of market opportunity and since currently different pharmaceutical marketing strategies adopted by various drug companies are too attractive in developing countries [28]. Inadequate enforcement of the pharmaceutical law was establish to be the leading contributing factor to irrational prescribing practice [29]. It keeps them moving and working as they freely. This gap allows physicians become involved in unethical activity. A study reported that the loss of credibility of physicians’ in the eyes of the patients and the public as a consequence of the nature and effect of the relationship between pharmaceutical companies [30]. Key informants also revealed that they were influenced by pharmaceutical marketing mix strategies.

Promotion as one of the strategies used by pharmaceutical companies 67.8, 73.5, 63.9 and 56.6% of physicians perceived that participating in company-sponsored CME, information from MRs, frequent visits of MRs, and information from promotional drug brochures was influenced their prescribing behavior respectively. This finding revealed that higher influence of physicians as compared to studies conducted in Lebanon, which mentioned that physicians participating in CME conferences, visits of MRs, and promotional drug brochures were influenced their prescribing pattern 39, 51.1, 18.7% respectively [31]. Also, a study conducted in Saudi Arabia reported that the frequency of visits MRs was 56.6% of physicians’ influence in their prescribing decisions [32].

The present study revealed that lower influence of physicians’ prescribing behavior as compared to the study conducted Addis Ababa; which stated that 44.2, 69.7, and 75.4% of physicians participating in company-sponsored CME, frequency of visits of MRs, and the information from MRs was influenced their prescribing behavior respectively [25]. Also, A study done in south-eastern city in United States of America reported that influenced physicians’ responses to marketing strategies by drug brochures were 68% and the visit of MRs was 73% [33].

The discrepancy among studies might be due to medical professionals after graduating from medical school have difficulties to update themselves because of limited sources of information. A study conducted at Hawassa University teaching and referral hospitals in southern Ethiopia reported that lack of drug information was one of the factors that lead to physicians’ irrationally prescribing medicines [34]. Because the source of information is limited, prescribers rely on the information they find on their environment. A review reported that identified that information provided by multinational companies is often biased and sometimes dangerously misleading [35]. This inappropriate use has serious health and economic consequences for the success of health care system at national level and adopting this information into clinical areas is too difficult [36].

The qualitative part of the present study indicated that the promotion strategies; visits of MRs, attending CME and drug brochures are the major source of drug information to their work. This finding was also supported by other studies done in Pakistan, which reported that physicians recognize MRs as information providers and beneficial patronage to their work [8].

In addition, the present study revealed that perceived influence of physicians on their prescribing behavior by invitation to visit a pharmaceutical manufacturing plant was 60.3%. this finding is comparable to the study conducted in Addis Ababa; which reported that 63.9% of physicians visit a pharmaceutical manufacturing plant was influenced their prescribing behavior [25].

This might be attributed to drug companies choose physicians in a serious way and take them to on a tour abroad to see manufacturing sites. Studies conducted in Pakistan also reported that attending pharmaceutical company-sponsored travels to touristic locations and visiting manufacturing plants have increased in the prescribing rate after the physicians attended a company-sponsored event with all their expenses covered [37]. This leads to incorrect generalization in physicians after each visit makes them think about that company. The qualitative finding also reported that tours and invitation to visit the pharmaceutical plant that helped physicians for strengthening their relationship with the company and had a contribution to changing their prescribing behavior.

Regarding to product strategy, this study found that 58.8, 54.4, and 80.9% of physicians’ perceived that country of pharmaceutical product manufacturer, form of delivery of the medicine, and quality of medicine was influenced their prescribing behavior respectively. Lower influences as compared to study conducted in Nairobi revealed that physicians influenced in their prescribing behavior by form of delivery of the medicine was 85.8% [24]. But higher influences as compared to study done in Saudi Arabia in which 46.2% of physicians influenced their prescribing decision by a source of the company that produces the drug [32]. The study conducted in Addis Ababa also reported that 34.5% of physicians’ quality of the medicine was influenced their prescribing behavior [25]. The difference among studies might be attributed to companies spend a lot of money every year for innovation and they come with better improvement with a condition on their dosage, indication, side effect and cost from the oldies one. Physicians select new generation and improved medication products after MRs told to them. Also, difficult to diagnose because there is no fully-equipped laboratory at the health facility in the town due to that they prescribe drugs having better coverage. As the government community health insurance scheme continues to grow in the town as a strategy for reducing financial catastrophic shock [38], physicians prescribe what is considered to be good quality because there is an insurance scheme that covers the cost of patients. Most communities nowadays have increased awareness of the importance of quality and will have also their own contribution.

The city is close to the harbor and no strict regulation done by Ethiopian food and medicine regulatory authority at the border areas makes a better supply of medicine with questionable quality of the medicine. In Dessie town, there are many pharmacies and wholesalers where medicines transactions are very active and it is difficult for doctors to identify counterfeit drugs from the original product. Due to this reason physicians preferred the known sources and good quality of medicines. China and India are the leaders in counterfeit drug production [39]. So, drugs come from those countries that are often perceived that has unproven quality. The qualitative finding reported that change in the preparation of the drug formulations, knowing the country where the drug is manufactured makes them think that the drug has a good quality and influence on their work when prescribing medicine to patients.

In the present study, 88.2, 64, and 50% of physicians perceived that pharmaceutical product availability, the inclusion of medicine in the hospital medicine list, and availability of local agents representing the principal company were influenced their prescribing behavior respectively. It was higher influences as compared to a study conducted in Nairobi stated that influenced physicians’ on prescribing behavior by medicine availability was 65.1%, availability of the medicine in hospital formularies was 45.4%, and local agent representing the principal company was 31.8% [24]. This might be due to various reasons, such as people move from one place to another for different reasons, the spread of the disease also increases in the town, leading to diseases are occurring that we have never seen before. Moreover, the climate difference between the towns and around the city increases the spread of disease and patients. To use this opportunity, companies will have an equal agent like Addis Ababa to fulfill this need. World Health Organization and Ethiopian pharmaceutical policy emphasized that each health facility to develop their own facility-specific medicine list, made the physicians forced to use the medical drugs that are available there. Key informants also revealed that improving the supply chain of pharmaceuticals had made their medical work easier and effect on them.

Regarding pricing strategy, the present study found that physicians’ perceived influence on their prescribing behavior by disclosure of actual price of the product was 68.4%, the price of the drug and effectiveness of therapy was 82.3%, and price competition among the pharmaceutical companies was 44.1%. This finding was higher than a study conducted in Addis Ababa, in which the influence of pricing of medicine to the physicians’ prescribing behavior was 23% [25]. The result was also higher influence as compared to study in Nairobi reported that 56.4% of physicians’ by price of the drug in relation to the severity of the indication was influenced their prescribing behavior and lower influences stated 81.6% of physicians by price in relation to competing product was influenced their prescribing behavior [24].

The difference among studies might be attributed according to the World Bank, Ethiopia lies in a lower-income country [40]. Although the purchasing power of the community varies, they prescribe medications with the consideration of patients’ wealth. The current medical system considers physicians and patients as the pillars of decision-maker deciding what treatment will begin for patients’ condition. Physicians compare the relative costs, effects of different types of drugs and estimate the strengths and weaknesses of alternatives to determine options to select and prescribe pharmaceutical products for patients [41]. The pharmaceutical policy in our country is making companies freely to change the price of their products as they need and different level of markup utilized by different pharmaceutical companies. Physicians comprise the least costly alternatives when the outcomes of two or more drugs are virtually the same. Despite the government regulation and insurance company’s guidelines might have its contribution to this variation. The qualitative findings reported that knowing the price of the drug and making a discount for patients has a benefit and influenced their prescribing behavior.

In this study, 61, 52.2, 58.8, and 52.2% of physicians perceived that promotion strategy, product strategy, price strategy and place strategy were influenced their prescribing behavior respectively and there is a statistically significant difference among marketing mix strategies (*p* = < 0.001). This might be due to different companies develop one marketing strategy with the other in a more efficient way, which makes it distinct to each other. The ultimate objective of the pharmaceutical marketer would be to devise a product that will be seen as different in the eyes of physicians. The pharmaceutical companies have four basic ingredients (promotion, place, product, and price) utilize to achieve a great market share [42]. Companies compete with each other for better profits by using different strategy will lead to more drug sales and increase market share in the town.

The qualitative part of the current study indicated that physicians who are working in public and private hospitals perceived influence by pharmaceutical marketing mix strategies on their prescribing behavior. This finding was also supported by other studies done in Yemen that reported that pharmaceutical companies and their marketing activities were effects on their prescribing behavior [43].

Regarding the determinants of influencing physicians’ prescribing behavior, a significant result was found only for two variables, working areas (*p* = 0.04) and specialty (*p* = 0.01). A change in physician’s specialty to ear, nose, and throat specialty will shift perceived influence by − 0.06. Changing working area from public or private sector will shift perceived influence by 0.15. This might be due to the health care system in the private sector is open; the pharmaceutical company MRs easily will meet individual doctors. In addition, although it is expected that physicians prescribe generic drugs to the patients but they mostly prescribe brand drugs. So, the promoter will easily find them. Patients visiting the private sector want better medicine to be prescribed for their condition. Currently, pharmaceutical companies are bringing their specific medications to the market for specific uses by modifying the preparation of drugs. During this time, they directly contact to specialized physicians to prescribe those medications. They choose a few selected specialists and make them prescribe more drugs on their prescriptions.

Availability of high pharmaceutical sectors operating within close proximity of one building is an important catalyst for growth among pharmaceutical companies as they benefit from the value chain that exists within a city. These make them focus physicians found in the city and keep them updated only on things currently available products. One of the factors that make the health care system not good in our country is irrational prescribing. The study conducted in Ethiopian referral hospitals reported that a mean number of drugs per prescription was 5.1 [44]. One of the reasons for this is the influence of pharmaceutical companies on physicians for the deterioration of the medical system. This leads to a reduction in the quality of pharmacotherapy, wastage of resources, high treatment cost, resistance to antibiotics, and making illness more serious [45].

Pharmaceutical companies have to work ethically if effective health care services required to be given to patients. Both the patient and the doctor need to make a decision in cooperatively to the treatment options. The regulatory agency in the country should make appropriate laws and implement them. It must be a functionalized drug therapeutic committee and drug information center on a private and public health facility. Physicians also need to refrain from unethical activity provided by pharmaceutical companies that are unnecessary and does not input scientific knowledge to their work.

### Practical implication of the study

There is a need for a good rational prescribing practice and delivery of the health care system in Dessie city. Consequently, the influence of pharmaceutical companies on physicians should be examined. The finding of the research is relevant for creating awareness on the influence of pharmaceutical marketing mix strategies on physicians’ prescribing behaviors. The results obtained in quantitative and qualitative studies were complimentary. This can help responsible stakeholders to formulate intervention to maximize rational prescribing medication in the delivery of health care services. In addition to the above points, the findings of this study also will give a clue to conduct further investigation in the area and evaluate the ethical practices of pharmaceutical marketing mix strategies.

### Strength and limitation of the study

This study assessed the influence of pharmaceutical marketing mix strategies on physicians prescribing behavior using both (qualitative and quantitative) study design. As a limitation, the present study was unable to determine the temporal effect of a company’s marketing strategy on physician prescribing behavior due to the cross-sectional nature of the study. To measure the influence of pharmaceutical marketing mix strategies on physicians’ prescribing behavior respondents were asked Likert type questions that were answered based on selecting an appropriate choice on a scale from a given list of activities performed as pharmaceutical marketing mix strategies. This prevents to measure the association of individual pharmaceutical marketing mix strategies with the influence of physicians prescribing behavior. Likert scale type questions fail to measure the true attitude (opinions) of physicians as the space between each choice (5-point scale) is not equaled distant. Moreover, respondent’s usually avoiding choosing extreme values on the scale. Hence, there is a central tendency error committed by our respondents related to the nature of the data collection tool. The findings of this study may not represent other health care facilities of the country, since it was conducted in the stated town and the issue of context may vary. The volunteer nature of this study may affect the findings of this project, such as it is difficult to know the extent to which the participants were truthful in the perceptions and opinions they share with PI or whether they were telling the PI the things they think the PI wants to hear.

## Conclusion

Pharmaceutical manufacturers try to influence physicians through a variety of strategies to increase their market share by inducing more prescriptions. More than half of physicians prescribe medication due to the influence of pharmaceutical manufacturers. Nearly two-thirds of physicians influenced their prescribing behavior by promotion strategy, product strategy, place strategy, and price strategy.

Participating in company-sponsored CME, frequency of visits and information from MRs, information from promotional drug brochures, and invitation to visit a pharmaceutical manufacturing plant, country of pharmaceutical product manufacturer, image of pharmaceutical company, quality of medicine, supportive evidence of the efficacy of the medicine given by pharmaceutical company, release of innovations of drugs from product strategy, pharmaceutical product availability, inclusion of medicine in the hospital medicine list, and availability of local agent representing the principal company, price of the drug and effectiveness of therapy, disclosure of actual price of the product, and price of medication with quality were strategies influenced most physicians in their prescribing behavior. Pharmaceutical companies target physicians based on their specialty and their working area.

The qualitative study generates some additional insights into the influence of pharmaceutical marketing mix strategies. Key informants revealed that they were influenced by pharmaceutical marketing mix strategies on their prescribing behavior and these insights obtained should be viewed as preliminary complimentary propositions that are not necessarily fully generalizable. All concerned stakeholders should work together to ensure a good health care system and drug use. Strengthening the regulation and maintaining ethical practice would help to rationalize the physicians’ prescribing practice.

## Supplementary Information

**Additional file 1.** Survey questionnaire, which includes: Information, consent sheets and Self-administered questionnaire for quantitative approach.

**Additional file 2.** Key informants interview guide; Information and consent sheets for qualitative approach.

**Additional file 3.** Ethical clearance and approval letter.

## Data Availability

All data generated or analyzed during this study are included and available in this published article.

## Abbreviations

MRs: Medical representatives
CME: Continual medical education
US$: United States of America Dollar

## References

[1] Kotler P, Armstrong G (2010). Principles of marketing. Pearson education.

[2] Ding M, Eliashberg J, Stremersch S (2016). Innovation and marketing in the pharmaceutical industry: springer.

[3] Ahmed RR, Khoso I, Kiyani P, Jeswani DD, Ahmed J, Vveinhardt J (2014). Marketing practices of Pakistan pharmaceutical industry and physician prescription behavior. World Appl Sci J.

[4] Armstrong G, Adam S, Denize S, Kotler P (2014). Principles of marketing. Pearson Australia.

[5] Ladeira W, Dalmoro M, Eduardo Maehler A, FalcãoAraujo C (2011). Drug prescription practices in Brazil: a structural equation model. Int J Pharm Healthc Mark.

[6] Parker RS, Pettijohn CE (2006). Pharmaceutical drug marketing strategies and tactics: a comparative analysis of attitudes held by pharmaceutical representatives and physicians. Health Mark Q.

[7] Vyas M, Panesar A (2019). Pharmaceutical marketing communication strategies and its influence on physician prescription preference. ZENITH Int J Multidisciplinary Res.

[8] Al-Areefi MA, Hassali MA (2013). Physicians’ perceptions of medical representative visits in Yemen: a qualitative study. BMC Health Serv Res.

[9] Al-Haddad MS, Hamam F, AL-Shakhshir SM. (2014). General public knowledge, perceptions and practice towards pharmaceutical drug advertisements in the Western region of KSA. Saudi Pharm J.

[10] Shaw B, Whitney P (2016). Ethics and compliance in global pharmaceutical industry marketing and promotion: the role of the IFPMA and self-regulation. Pharm Policy Law.

[11] Bansal RK, Das S (2005). Unethical relationship between doctors and drugs companies. J Indian Acad Forensic Med.

[12] Brezis M (2008). Big pharma and health care: unsolvable conflict of interests between private enterprise and public health. Isr J Psychiatry Relat Sci.

[13] Manchanda P, Honka E (2005). The effects and role of direct-to-physician marketing in the pharmaceutical industry: an integrative review. Yale J Health Pol'y L & Ethics.

[14] Wazana A (2000). Physicians and the pharmaceutical industry: is a gift ever just a gift?. Jama.

[15] Sutton J, Kellow N (2011). An enterprise map of Ethiopia-Chinese version: international growth Centre.

[16] FMOH (2003). Assessment of the pharmaceutical sector in Ethiopia.

[17] FMOH (2015). National strategy and plan of action for pharmaceutical manufacturing development in Ethiopia (2015–2025).

[18] EFDA (2010). Food medicine and health care administration and control proclamation 661/2009. Federal Negarit Gazette.

[19] Kotler P (2012). Kotler on marketing. Simon and Schuster.

[20] Datta A, Dave D (2017). Effects of physician-directed pharmaceutical promotion on prescription behaviors: longitudinal evidence. Health Econ.

[21] Gebreegziabher S (2017). Prescription drug promotion and prescribing behavior of physicians’ in case of Addis Ababa green licensed private hospitals.

[22] Workneh BD, Gebrehiwot MG, Bayo TA, Gidey MT, Belay YB, Tesfaye DM, Kassa TT (2016). Influence of medical representatives on prescribing practices in Mekelle, Northern Ethiopia. PloS One.

[23] Sayandhan T, Kodithuwakku SS, Gunaratne LH (2008). Influence of marketing mix in prescribing pharmaceutical products by ophthalmologists in Sri Lanka.

[24] Gichoch M (2006). Influence of marketing mix on medical doctors’ choice of prescription drugs in Nairobi.

[25] Dogramatzis D (2002). Pharmaceutical marketing: a practical guide: CRC Press.

[26] Katzung BG (2018). Rational Prescribing & Prescription Writing. Basic & Clinical Pharmacology.

[27] Risk R, Naismith H, Burnett A, Moore SE, Cham M, Unger S (2013). Rational prescribing in paediatrics in a resource-limited setting. Arch Dis Childhood.

[28] Kalotra A (2014). Marketing strategies of different pharmaceutical companies. J Drug Delivery Therapeutics.

[29] Suleman S, Woliyi A, Woldemichael K, Tushune K, Duchateau L, Degroote A (2016). Pharmaceutical regulatory framework in Ethiopia: a critical evaluation of its legal basis and implementation. Ethiop J Health Sci.

[30] Campbell EG, Rao SR, DesRoches CM, Iezzoni LI, Vogeli C, Bolcic-Jankovic D, Miralles PD (2010). Physician professionalism and changes in physician-industry relationships from 2004 to 2009. Arch Int Med.

[31] Khazzaka M (2019). Pharmaceutical marketing strategies’ influence on physicians’ prescribing pattern in Lebanon: ethics, gifts, and samples. BMC Health Serv Res.

[32] Ibrahim IAY, Bélanger CH (2015). Pharmaceutical representatives an d prescription decisions by physicians in Saudi Arabia. J Marketing Manag.

[33] Spiller LD, Wymer WW (2002). Physicians’ responses to marketing strategies of pharmaceutical companies. J Pharm Marketing Manage.

[34] Desalegn AA (2013). Assessment of drug use pattern using WHO prescribing indicators at Hawassa University teaching and referral hospital, south Ethiopia: a cross-sectional study. BMC Health Serv Res.

[35] Goldacre B (2014). Bad pharma: how drug companies mislead doctors and harm patients: Macmillan.

[36] World Health Organization. Medicines use in primary care in developing and transitional countries: fact book summarizing results from studies reported between 1990 and 2006: World Health Organization; 2009. Available at:https://apps.who.int/iris/bitstream/handle/10665/70032/WHO_EMP_MAR_2009.3_eng.pdf.

[37] Masood I, Ibrahim MIM, Hassali MAA, Ahmad M, Mansfield PR (2012). Evaluation of pharmaceutical industry–sponsored educational events attended by physicians in Pakistan. J Med Mark.

[38] Alebachew A, Yusuf Y, Mann C, Berman P (2015). Ethiopia’s progress in health financing and the contribution of the 1998 health care and financing strategy in Ethiopia.

[39] Vishnuvardhan C, Srinivas R, Satheeshkumar N (2014). Development and validation of a UPLC method for screening potentially counterfeit anti-hypertensive drugs using design of experiment. Anal Methods.

[40] World Bank. The World Bank in Ethiopia. Accessed 23 Dec 2019. Available at: https://www.worldbank.org/en/country/ethiopia/overview2019.

[41] Rascati K (2013). Essentials of pharmacoeconomics: Lippincott Williams & Wilkins.

[42] Bee AH (2009). Market share strategies in the pharmaceutical industry. Unitar e-journal..

[43] Al-Areefi MA, Hassali MA (2013). The role of pharmaceutical marketing and other factors in prescribing decisions: the Yemeni experience. Res Soc Adm Pharm.

[44] Sada O (2017). Irrational use of medications among elderly patients in an Ethiopian referral hospital. Afr J Pharm Pharmacol.

[45] Chapman S, Durieux P, Walley T (2004). Good prescribing practice. Mossialos et al.

