# Supplementary material for: Influence of pharmaceutical marketing mix strategies on physicians’ prescribing behaviors in public and private hospitals, Dessie, Ethiopia: a mixed study design
Source: BMC Public Health. 2021 Jan 7;21:65. doi: 10.1186/s12889-020-10063-2 (PMC7791818; doi:10.1186/s12889-020-10063-2)
Supplement: Supplementary file 1 — Additional file 1. Survey questionnaire, which includes: Information, consent sheets and Self-administered questionnaire for quantitative approach. [file 12889_2020_10063_MOESM1_ESM.docx]

**WOLLO UNIVERSITY**

**COLLEGE OF MEDICINE AND HEALTH SCIENCES**

**DEPARTMENT OF PHARMACY**

## Information Sheet for the quantitative part

**Dear Respondent:**

My name is Abel Demerew a Master‘s student at Wollo University, College of Health Sciences, School of social and administrative Pharmacy, Department of Pharmacy.

I kindly request you to participate in a study that is aimed to collect information on the **“Influence of pharmaceutical marketing mix strategies on physicians’ prescribing behaviors in hospitals, Dessie, Ethiopia”.** The true answer that you give to me means a lot to achieve the goal of the research. I want to ask you about different things relating to your work activities. The information will be kept strictly confidential you provide. Any information related to your identifier, you will ever be realized by an outsider will never be used on the research. Participation in this survey is completely voluntary. However, I encourage you to participate in this study since the results will help a lot for different public health interventions.

## Consent sheet for the quantitative part

I, the undersigned, understand the nature of the study, benefits, and my right to voluntary participation, confidentiality, and withdrawal from the study without any victimization. I have had the opportunity to ask questions and answered to my satisfaction.

I now freely consent to take part in this study.

Signature of the participant_______________

Date___________________________

Data collector

Name __________________________ signature_________ Date_____/_______/____E.C.

Your participation will be greatly appreciated.

Yours Faithfully,

## Self-administered questionnaire

**Part I: Socio-demographic Characteristics**Please tick and input your personal details information under the following headings.

| 1.1 | Sex | 1. Male……….. 2. Female ……….. | |
| --- | --- | --- | --- |
| 1.2 | Age |  | |
| 1.3 | Education | 1. General practitioner 2. Specialized | |
| 1.4 | Specialty(skip to question 1.6 if you had not specialized yet) | 1. Internist…………… 2. Surgeon…………… 3. Gynecologist………… 4. Pediatrician…………... | 1. Dermatologist……….. 2. Orthopedics………….. 3. Resident……………... 4. ENTs………………. |
| 1.5 | Country of specialization | 1. Ethiopia …………….. 2. Outside Ethiopia……………. | |
| 1.6 | Country of first degree education | 1. Ethiopia …………. 2. Outside Ethiopia …………. | |
| 1.7 | Years of experience | ……………. | |
| 1.8 | Working areas | 1. Public……… 2. Private……… 3. Both …….. | |

**Part 2: Questions related to effect of promotion on prescribing behavior**

Here is a list of promotional tools used by pharmaceutical companies. Please circle one category of each promotional tool depending on whether that tool motivates you to select and prescribe a certain product of a drug company. Please rate accordingly

| **No** | **Description** | **Strongly agree**  **(1)** | **Agree**  **(2)** | **Neutral**  **(3)** | **Disagree**  **(4)** | **Strongly disagree**  **(5)** |
| --- | --- | --- | --- | --- | --- | --- |
| 2.1 | Participating in company-sponsored continual medical education | 1 | 2 | 3 | 4 | 5 |
| 2.2 | Information from medical representative | 1 | 2 | 3 | 4 | 5 |
| 2.3 | Frequently visits of medical representative | 1 | 2 | 3 | 4 | 5 |
| 2.4 | Sales calls made by pharmaceutical companies | 1 | 2 | 3 | 4 | 5 |
| 2.5 | Free drug samples given by pharmaceutical company | 1 | 2 | 3 | 4 | 5 |
| 2.6 | Information from promotional drug brochures | 1 | 2 | 3 | 4 | 5 |
| 2.7 | Different gifts from pharmaceutical company | 1 | 2 | 3 | 4 | 5 |
| 2.8 | Participating pharmaceutical company-sponsored entertainments/recreational events | 1 | 2 | 3 | 4 | 5 |
| 2.9 | Sponsorship for travel in conference | 1 | 2 | 3 | 4 | 5 |
| 2.10 | Subscription of journals with direct mail | 1 | 2 | 3 | 4 | 5 |
| 2.11 | Invitation to visit a pharmaceutical manufacturing plant | 1 | 2 | 3 | 4 | 5 |
| 2.12 | Personal relationship to company | 1 | 2 | 3 | 4 | 5 |
| 2.13 | Product launch meeting | 1 | 2 | 3 | 4 | 5 |
| 2.14 | Public relation of pharmaceutical company | 1 | 2 | 3 | 4 | 5 |

**Part 3: Questions related to effect of product on prescribing behavior**

Here is a list of product strategy used by pharmaceutical companies. Please circle one category depending on whether that activity motivates you to select and prescribe a certain product of a drug company. Please rate accordingly

| **No** | **Description** | **Strongly agree**  **(1)** | **Agree**  **(2)** | **Neutral**  **(3)** | **Disagree**  **(4)** | **Strongly disagree**  **(5)** |
| --- | --- | --- | --- | --- | --- | --- |
| 3.1 | Country of pharmaceutical product manufacturer | 1 | 2 | 3 | 4 | 5 |
| 3.2 | Image of pharmaceutical company | 1 | 2 | 3 | 4 | 5 |
| 3.3 | Supportive evidence of the efficacy  of the medicine given by pharmaceutical company | 1 | 2 | 3 | 4 | 5 |
| 3.4 | Release of new innovations or combinations  of drugs | 1 | 2 | 3 | 4 | 5 |
| 3.5 | Form of delivery of the medicine | 1 | 2 | 3 | 4 | 5 |
| 3.6 | Easy to remember brand names | 1 | 2 | 3 | 4 | 5 |
| 3.7 | Reputation of the source of medicine | 1 | 2 | 3 | 4 | 5 |
| 3.8 | Quality of medicine | 1 | 2 | 3 | 4 | 5 |
| 3.9 | Fixed-dose packaging of the product | 1 | 2 | 3 | 4 | 5 |
| 3.10 | Full therapy packaging | 1 | 2 | 3 | 4 | 5 |

**Part 4: Questions related to effect of place (distribution) on prescribing behavior**

Here is a list of distribution strategy used by pharmaceutical companies. Please circle one category depending on whether that activity motivates you to select and prescribe a certain product of a drug company. Please rate accordingly

| **No** | **Description** | **Strongly agree**  **(1)** | **Agree**  **(2)** | **Neutral**  **(3)** | **Disagree**  **(4)** | **Strongly disagree**  **(5)** |
| --- | --- | --- | --- | --- | --- | --- |
| 4.1 | Pharmaceutical product availability | 1 | 2 | 3 | 4 | 5 |
| 4.2 | Inclusion of medicine in the hospital medicine list | 1 | 2 | 3 | 4 | 5 |
| 4.3 | Availability of local agent (importer/distributor) representing the principal company | 1 | 2 | 3 | 4 | 5 |
| 4.4 | Availability of real-time product information from distribution intermediaries | 1 | 2 | 3 | 4 | 5 |
| 4.5 | Presence of sole supplier | 1 | 2 | 3 | 4 | 5 |
| 4.6 | Fast deliveries with special storage and distribution of medicines | 1 | 2 | 3 | 4 | 5 |
| 4.7 | Reverse pharmaceutical (product recall) | 1 | 2 | 3 | 4 | 5 |

**Part 5: Questions related to effect of price on prescribing behavior**

Here is a list of pricing strategy used by pharmaceutical companies. Please circle one category depending on whether that activity motivates you to select and prescribe a certain product of a drug company. Please rate accordingly

| **No** | **Description** | **Strongly agree**  **(1)** | **Agree**  **(2)** | **Neutral**  **(3)** | **Disagree**  **(4)** | **Strongly disagree**  **(5)** |
| --- | --- | --- | --- | --- | --- | --- |
| 5.1 | Disclosure of actual price of the product | 1 | 2 | 3 | 4 | 5 |
| 5.2 | Price discounts technique for the product | 1 | 2 | 3 | 4 | 5 |
| 5.3 | Price of the drug and effectiveness of therapy | 1 | 2 | 3 | 4 | 5 |
| 5.4 | Price of medication in relation to quality | 1 | 2 | 3 | 4 | 5 |
| 5.5 | Price competition among pharmaceutical company | 1 | 2 | 3 | 4 | 5 |
| 5.6 | Price for full course therapy | 1 | 2 | 3 | 4 | 5 |
