# Supplementary material for: Influence of pharmaceutical marketing mix strategies on physicians’ prescribing behaviors in public and private hospitals, Dessie, Ethiopia: a mixed study design
Source: BMC Public Health. 2021 Jan 7;21:65. doi: 10.1186/s12889-020-10063-2 (PMC7791818; doi:10.1186/s12889-020-10063-2)
Supplement: Supplementary file 2 — Additional file 2. Key informants interview guide; Information and consent sheets for qualitative approach. [file 12889_2020_10063_MOESM2_ESM.docx]

**WOLLO UNIVERSITY**

**COLLEGE OF MEDICINE AND HEALTH SCIENCES**

**DEPARTMENT OF PHARMACY**

## Information and consent sheet for the qualitative part

**Dear Respondent:**

My name is Abel Demerew a Master‘s student in social and administrative pharmacy at Wollo University, Department of pharmacy.

I kindly request you to participate in a study that is aimed at **“Influence of pharmaceutical marketing mix strategies on physicians’ prescribing behaviors in hospitals, Dessie, Ethiopia”.** The result of the study will be valuable to concerned stakeholders and decision-makers to intervene and promote rational prescribing.The true answer that you give to me means a lot to achieve the goal of the research. I want to ask you about different things relating to your work activities.

The interview will take 30 minutes and recorded. A transcript will be produced and you will be sent the transcript and allowed to correct any factual errors. The transcript will be produced by the principal investigator. Access to the transcript will be limited to the principal investigator and academic colleagues. Any data from the interview and direct quotation made available through academic publication or other means will anonymize so that you cannot be identified. At any time you may also stop the interview completely without any consequences at all. However, your participation will be greatly appreciated.

Are you willing to continue the interview?

Yes No

If yes the interview will continue

## Interview guide for key informants

**Part 1: Background information.**

| **1** | Age………………. | **4** | Current position……………….. |
| --- | --- | --- | --- |
| **2** | Sex………………. | **5** | Years of experience…………….. |
| **3** | Educational level……………… |  | |

**Part 2: Interview guide related to pharmaceutical marketing mix strategies affecting prescribing behavior.**

1. How do you see pharmaceuticals supply status for current community health problems and the cost of the pharmaceuticals in terms of community income?
2. Currently, pharmaceutical manufacturers use different marketing strategies to get doctors and how do they try to communicate to you?
3. Currently, the pharmaceutical company utilizes different marketing strategies to convince physicians –

**Promotion strategy -** Participating in company-sponsored continual medical education, information from medical representatives, frequency of visits, sales calls made by companies, free drug samples, promotional drug brochures, different gifts, company-sponsored entertainments/recreational events, subscription of journals with direct mail, Invitation to visit a manufacturing plant and relation with the company.

**Product strategy -** Country of manufacture of pharmaceutical product, image of company, new innovations or combinations of drugs, easy to remember brand names and form of delivery of the medicine.

**Price strategy -** Disclosure of actual price of the product, Price discounts by a competing manufacturer, price of the drug and effectiveness of therapy.

**Place strategy -** Making the drug available at the health facility and soon, availability of local agent (importer/distributor) representing the principal company and with fast deliveries of medicines.

- 1. How can these techniques benefit you?
  2. Do you think this manufacturer activity has any influence on your work, when you are prescribing medication? How? Can you tell me an example?

1. Currently, pharmaceutical manufacturers use different marketing strategies to increase their market share. How would you evaluate this activity?

**ወሎዩኒቨርሲቲየጤናእናህክምናሳይንስኮሌጅፋርማሲክፍል**

**ክፍል፤1 ሀኪሞችንቃለመጠይቅለማድረግየፈቃደኝነትመጠየቂያቅፅ**

**መግቢያ**

ስሜአቤልደምረዉይባላል፡፡ በወሎዩኒቨርሲቲየፋረማሲትምህርትክፍልየሶሻልናአድሚንስትሬቲቭፋርማሲተማሪስሆንየመድሃኒትአምራቾችበሃኪሞችየመደሃኒትአስተዛዘዝስርአትላይያላቸዉንተጽእኖበማጥናትላይእገኛለሁ፡፡ የጥናቱዉጤትለተለያዩባለድርሻአካላትናዉሳኔሰጪዎችየመፍትሄሀሳብለማስቀመጥናአግባባዊየመድሃኒትአጠቃቀምእዲኖርለማድረግይጠቅማል፡፡

ቃለመጠይቁ 30 ደቂቃየሚወስድሲሆንቃለመጠይቅለማድርግየድምጽመቅጃየምጠቀምይሆናል፡፡ ቃለመጠይቁወደወረቀትቅጂየሚቀየርሲሆንእረስዎበቅጂዉወቅትየሚፈጠሩስህተቶችንእንዲያርሙይደረጋል፡፡ የወረቀትቅጂየሚጻፈዉበዋናዉአጥኒሲሆንየቅጂዉዉጤትከዋናዉአጥኒናከዩኒቨርሲቲዉየምርምርአካላትዉጪዉስንይሆናል፡፡ ማናቸዉምየቃለመጠይቁዉጤቶችበህትመትናበተለያዩመንገዶችሲቀርቡየእረስዎንማንነትይፋየማያደርግይሆናል፡፡ በየትኛዉምጊዜቃለመጠይቁንየማቆምስልጣንአለዎት፡፡ እረስዎበጥናቱእንዲሳተፉአበረታታለሁ፡፡

ጥናቱላይለመሳተፍፈቃደኛነዎት

አዎ……..አይደለሁም………..

**መልሰዎአዎከሆነቃለመጠይቁይቀጥላል**

**ክፍል፤ 2 የተጠያቂዎችመረጃ**

1. እድሜ………2- ፆታ………3- የትምህርትደረጃ……………
2. የስራድርሻ……………...5- የስራልምድ…………………

**ክፍል፤ 3 የቃለመጠይቅጥያቄዎች**

1. በአሁኑጊዜላሉትየማህበረሰብጤናችግሮችየመድሀኒትአቅርቦትሁኔታውእንዲሁምየመድሀኒትአምራቾችየሚያቀርባቸውመድሀኒቶችየመሸጫዋጋቸውከህብረተሰቡገቢአንጻርአንዴትታየዋለህ?
2. በአሁንጊዜየመድሀኒትአምራቾችየተለያዩየገበያሰትራቴጅይጠቀማሉሀኪሞችንለማግኘትይሁንእናበአሁኑግዜእናነተንበምንአይነትመልኩለማግኘትይሞክራሉ?
3. የመድሀኒትአምራቾችገበያቸዉንለመጨመርከሚከተሎአቸውስትራቴጂውችውስጥ፡-**የመድሀኒትማስተዋወቅስትራቴጂ ፡** በመድሀኒትአምራቾችበሚዘጋጁየክህሎትማሳደጊያመድረኮችላይመሳተፍ፤ ከመድሀኒትአስተዋዋቂጋርያለዉየግንኙነትመጠንእናየሚገኝመረጃ፤ በአምራቾችየሚደረግጥሪ፤ የሚሰጥነጻየመድሀኒትማስተዋወቂያ፤ በራሪወረቀቶች፤ የሚሰጡስጦታዎች፤ የመዝናኛዝግጅቶች፤ በመረጃቋትየሚለቀቁመረጃዎች፤ የመድሀኒትማምረቻጉብኝትእናከአምራቾችጋርያለግንኙነት፡፡

**የመድሀኒትምርትሰትራቴጂ፡** የመድሀኒትአምራቹየሚገኝበትሀገር፤ ያለውገጽታ፤ አዲስምርቶችንማምረታቸው ፤ የተለያዩመድሀኒቶችንበአንድላይማጣመራቸው፤ ወደገበያየሚቀርብበትመንገድእናየመድሀኒቱንየንግድስምለማስታወስቀላልማድረጋቸው፡፡

**የዋጋስትራቴጂ፡-** የመድሃኒቱንየሚሸጥበትንዋጋማሳወቃቸው፤ከተፎካካሪአምራችቅናሸማድረጋቸውእንዲሁምየመድሃኒቱየሚሸጥበትዋጋናየመፈወስአቅሙተመጣጣኝማድረጋቸው፡፡

**የቦታ (ማሰራጪያ) ስትራቴጂ፡-**መድሀኒቱበጤናተቋምውስጥእናበቅርቡእንዲገኝማድረጋቸው፤ ብቸኛወኪልወይምአቅራቢመኖሩእንዲሁምፈጣንአቅርቦትማድረጋቸው፡፡

- 1. እነዚህዘዴዎችለእናተስራበምንመልኩሊጠቀምዎትይችላል?
  2. ይህየመድሀኒትአምራቾችተግባርከእርሰዎስራላይማለትምመድሀኒትበሚያዙበትወቅትተጽኖአለውብለውያስባሉ? እንዴት?ምሳሌለነግሩኝይችላሉ?

1. በአሁኑግዜየመድሀኒትአምራቾችየተለያዩየገበያሰትራቴጅይጠቀማሉገበያቸዉንለመጨመር፡፡ ይሄንእንቅስቃሴቸውንአንዴትይገመግሙታል?
