# Supplementary figures and images for: Influence of pharmaceutical marketing mix strategies on physicians’ prescribing behaviors in public and private hospitals, Dessie, Ethiopia: a mixed study design
Source: BMC Public Health. 2021 Jan 7;21:65. doi: 10.1186/s12889-020-10063-2 (PMC7791818; doi:10.1186/s12889-020-10063-2)

**Ethical clearance and approval letters**

**
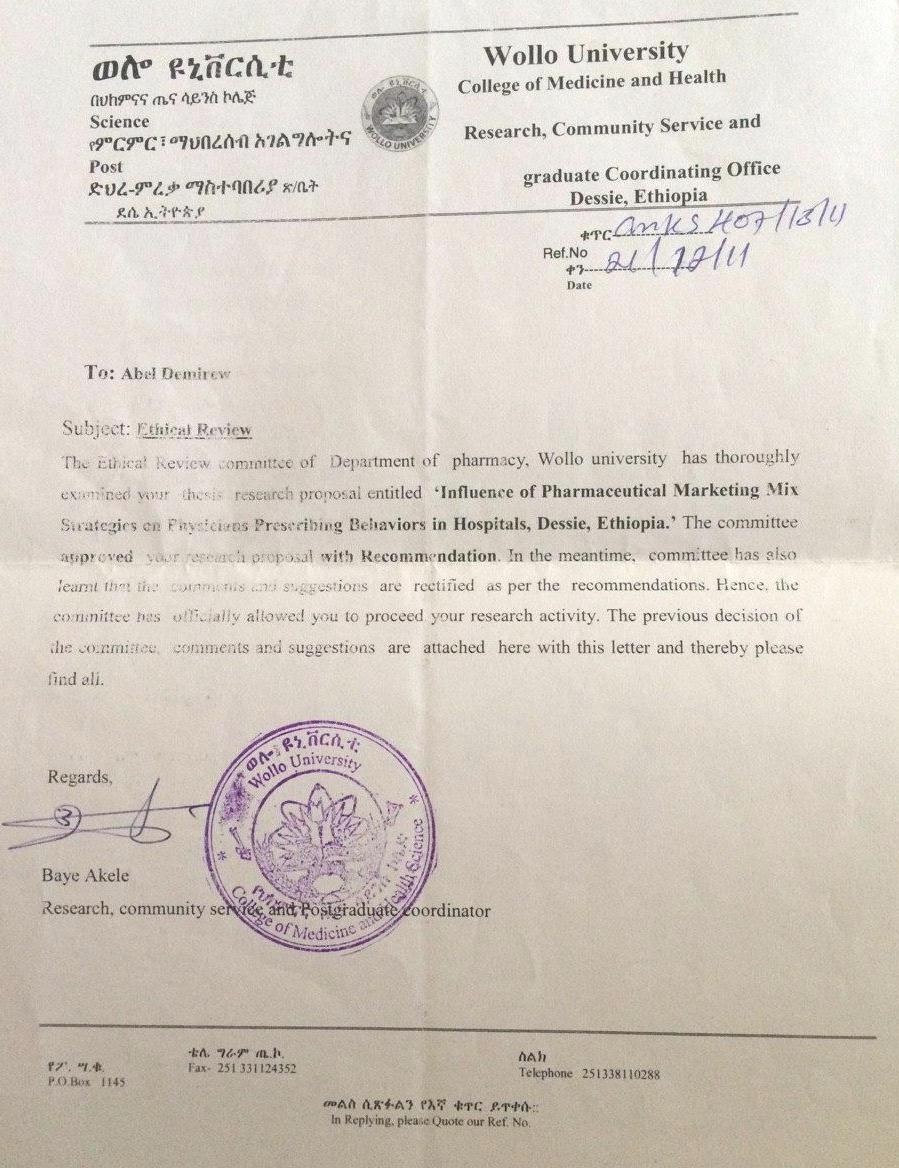
**

Supplement: Supplementary file 3 — Additional file 3. Ethical clearance and approval letter. [file 12889_2020_10063_MOESM3_ESM.docx]
